# Endogenous iron biomineralization in the mouse spleen of metabolic diseases

**DOI:** 10.1016/j.fmre.2024.07.004

**Published:** 2024-07-25

**Authors:** Ruowen Guo, Lei Zhang, Dongsheng Song, Biao Yu, Chao Song, Hanxiao Chen, Wenjing Xie, Chuanlin Feng, Guofeng Cheng, Kejun Hu, Jialiang Jiang, Zhe Qu, Haifeng Du, Xin Zhang

**Affiliations:** a High Magnetic Field Laboratory, CAS Key Laboratory of High Magnetic Field and Ion Beam Physical Biology, Hefei Institutes of Physical Science, Chinese Academy of Sciences, Anhui 230031, China; b Science Island Branch of Graduate School, University of Science and Technology of China, Anhui 230031, China; c Institutes of Physical Science and Information Technology, Anhui University, Anhui 230031, China

**Keywords:** Iron accumulation, Endogenous iron biomineralization, Nanoparticles, Metabolic diseases, Gradient high static magnetic fields

## Abstract

As the most abundant essential trace element in our human bodies, iron plays essential roles in multiple physiological and pathological conditions, including oxygen transport, cancer, diabetes, Alzheimer's and Parkinson's diseases. Although it has been shown that iron can form particles in some bacteria, migrating birds and fish, whether iron biomineralization can also occur in mammals is still debated. Here we demonstrate that iron metabolism abnormalities in diseased mice can directly lead to endogenous iron biomineralization and particle formation in the spleen. Excessive iron accumulation leads to endogenous particle formation with an averaged size of ∼800 nm. In the spleens of severely diabetic mice, they accumulate in the red pulp region, where the red blood cells are recycled. This directly changes the magnetic property of the spleens and results in spleen damage in a gradient high magnetic field. Therefore, our findings not only demonstrate the existence of endogenous iron biomineralization in metabolic diseases, but also set up a direct link among iron accumulation, magnetic property and magnetic responses of animal organs, which may also be applied in other iron metabolism-related diseases including cancer and Alzheimer's disease.

## Graphical abstract

**Figure:**
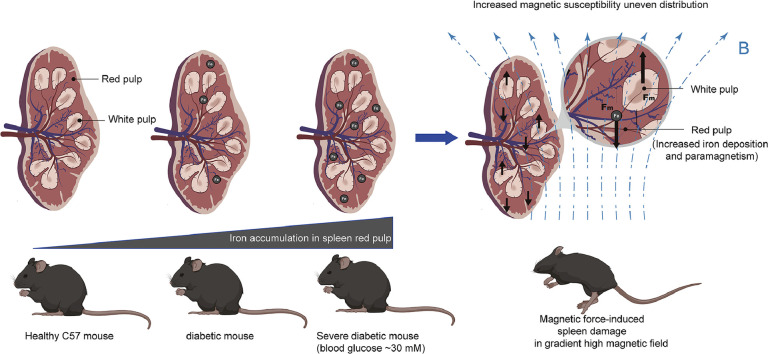

## 1 Introduction

Magnetite biomineralization as magnetosome by magnetotactic bacteria has been clearly demonstrated [1,2]. In some other species, such as honeybees, birds and fish, the existence of magnetite was also proved [[3], [4], [5]], although people argue about their potential function as magnetoreceptors [[6], [7], [8], [9]]. In contrast, in mammals, whether magnetite biomineralization intrinsically exists is still debated. In 1992, Kirschvink et al. showed that magnetite biomineralization can form in the human brain [10], but in 2016, these magnetic particles were proven to originate from air pollution, which entered the brain via the olfactory bulb [11]. In 2022, a study further identified a possible translocation pathway of various inhaled exogeneous particles from the lung to the brain tissue through blood circulation [12], which supports the external origin of the magnetite found in the brain but not endogenous biomineralization. However, it is interesting that another recent report shows that 11 prokaryote biomineralization genes are actually universally present among a diverse set of eukaryote taxa [13], which illustrates the possibility of conserved magnetite biomineralization pathways in not only bacteria but also higher eukaryotes.

On the other hand, iron metabolism has emerged as a key process that regulates multiple physiological and pathological conditions in our bodies. Iron metabolism is usually tightly regulated during physiological processes, whose deregulation has been linked to multiple diseases [14,15]. For example, iron overload has been demonstrated to be associated with increased cancer incidence and risk, while iron plays confirmed roles in cancer initiation, tumor growth and metastasis [16,17]. Iron overload is also considered as a risk factor for diabetes, and plays a direct and causal role in diabetes pathogenesis [18,19]. Moreover, iron metabolism also plays an essential role in the immune system [[20], [21], [22]]. However, whether iron accumulation in living animals can lead to iron biomineralization has not been reported.

Here, we show that excessive iron accumulation in metabolic diseases can lead to endogenous iron biomineralization and particle formation in the mouse spleen. In severe diabetes, the high level of iron leads to endogenous iron biomineralization and particle formation in the red pulp of the mouse spleen, which leads to unevenly distributed magnetic susceptibility and consequent changes in magnetic field sensitivity.

## 2 Materials and methods

### 2.1 Animal models and sample preparation

All procedures involving animals were approved by the Biomedical Ethics Committee of Anhui Medical University. 37 C57BL/6 J mice (5 weeks old) were purchased from the Nanjing GemPharmatech Co., Ltd. (Nanjing, China) and raised in ventilated cages in a temperature- and humidity-controlled room with a 12-hour light/dark cycle. C57BL/6 J mice were randomly allocated into healthy, diabetic, and severely diabetic groups. The diabetic group mice were administered intraperitoneal injections of streptozotocin (STZ) (0.01 M citrate buffer, pH 4.2) for five consecutive days (50 mg/kg body weight) and then kept for one week. A fasting blood glucose level of ≥ 16.7 mM was deemed successful in establishing the diabetic mouse model [23]. The severely diabetic group mice were given a single intraperitoneal injection of STZ at a dose of 150 mg/kg body weight and were kept for one week, with a fasting blood glucose level of ≥ 30.0 mM. All three groups were fed until they were 7 weeks old. Iron-overload mice were administered 1 mg iron (from Ferrous sulfate)/kg body weight mass dissolved in 150 µL 0.9 % saline solution by gavage daily for 10 weeks.

All mice were anesthetized with pentobarbital sodium (100 mg/kg body weight) via intraperitoneal injection and euthanized by exsanguination from the orbital plexus. The heart, liver, spleen, lung, kidney, and tumor tissues were then removed using ceramic scissors and forceps, and were placed in pre-cooled 0.1 M phosphate buffered saline (PBS) for immediate experimentation. The remaining tissues were stored in 4 % paraformaldehyde (PFA) or the −80 °C freezer until the next experimental step. No metal was used throughout the whole experimental procedure to avoid contamination.

### 2.2 *Superconducting quantum interference device* (*SQUID) measurement*

The Quantum Design magnetic property measurement system 3 (MPMS3) SQUID magnetometer was used to assess the magnetic properties of organ tissues obtained from mice, as well as water and FeCl_3_ solution. Prior to experimentation, the fresh tissue samples were immersed in PBS and kept on ice. The samples were then loaded into a sealed liquid sample holder (C130D; Quantum Design), which was subsequently vacuum-sealed. The M-H (magnetization-magnetic field strength) curve was measured at 300 K, with a magnetic field scanning range of −10,000 Oe to 10,000 Oe, and measurements were taken every 2500 Oe. For the H-T (magnetization-temperature) curve measurement, a magnetic field strength of 2000 Oe was employed, and the temperature was raised from 2 K to 300 K at a rate of 10 K/min, with measurements taken every 10 K. The magnetic moment signal of the sample was obtained after deducting the magnetic moment signal of the empty sample tube. The degree to which a material can be magnetized in an external magnetic field was calculated as the mass susceptibility (*χ*) using the equation

(1) $$\chi_{\left(\text{mass}\right)}=M/H$$

where *M* is the magnetization (moment per unit mass in the material), and *H* is the magnetic field strength. We divided the magnetic moment value of each measurement point by the mass of the sample to obtain the magnetization M per unit mass of the sample, and then used SquidLab software to perform a linear fit on all M vs. H data to obtain the fitted M-H curve, and the slope obtained is the magnetic susceptibility value of the sample [24]. For M-H curves, four data points were collected for each sample under each non-zero magnetic field (six data points under zero field), and M-H curves were obtained after fitting.

### 2.3 Laser ablation inductively coupled plasma mass spectrometry (LA-ICP-MS) analysis

LA-ICP-MS analysis was conducted utilizing a RESOlution laser ablation system (Applied Spectra, USA) coupled to a lasmaQuant MS Series ICP-MS (AnalytikJena, Germany). The spleen tissue of severely diabetic mice was cut into slices with a thickness of about 10 µm. The slices were made into cryostat sections and then dried completely. For the imaging studies, a systematic ablation of the entire spleen was executed with a laser spot measuring 50 µm in diameter, deploying an energy density of 2 J/cm², operating at a frequency of 10 Hz and a scanning speed of 50 µm/s, and high-purity helium is used as the carrier gas. Data processing was performed post-acquisition using the Iolite v4 software, and National Institute of Standards and Technology (NIST) 610 was used as an external standard to conduct semi-quantitative analysis of elemental content.

### 2.4 Superconducting magnet exposure

A 100 mm diameter room temperature bore superconducting magnet (Xi'an Superconducting Magnet Technology, China) was utilized to generate a vertical static magnetic field (SMF) with a central uniform magnetic field intensity of 9.4 T and a gradient SMF offset from the center. A sham group was established under identical conditions, including temperature, size, and air circulation, to serve as a comparison. The mice were placed on the off-center layers of the superconducting magnet, where the magnetic field exposure condition is ∼9 T, 10–20 T/m. The magnetic field exposure time lasted for a total of 14 h (2-hour field ascent, 10.5-hour constant from 18:00 to 08:30 overnight, and 1.5-hour descent).

### 2.5 HE staining

The tissues obtained from the mice were fixed in 10 % neutral buffered formalin for more than 24 h. After fixation, the tissues were dehydrated using ethanol concentrations and then cleared with xylene. The dehydrated tissue was embedded in paraffin and sectioned. Then the sections were immersed in xylene for 5 min to remove wax. They were then immersed twice in 100 % ethanol for 3 min each time and subsequently transferred to 95 % and 70 % ethanol for 2 min. After washing, the cells were stained with hematoxylin and eosin for the nucleus and cytoplasm staining. Finally, the slices were sealed with coverslips and examined under a Nikon TS100 microscope (Nikon, Japan).

### 2.6 DAB-enhanced Perls staining

Tissue sections were subjected to an incubation process employing a freshly prepared Perls solution consisting of 2 % potassium ferrocyanide and 2 % hydrochloric acid in an equimolar ratio for a duration of 40 min. Subsequently, the sections were subjected to a triple washing step using 0.1 M PBS. Following this, the sections underwent a rinse with PBS and a subsequent dehydration process utilizing an ethanol gradient. Subsequent to dehydration, the sections were subjected to a 5-minute incubation in DAB, followed by counterstaining with hematoxylin. The resultant sections were then dehydrated, mounted, and meticulously examined under a microscope (Nikon, Japan).

### 2.7 Masson's trichrome staining

The tissue sections were deparaffinized and rehydrated in distilled water before they were stained in modified Weigert's iron hematoxylin (stored in the dark) for 5 min. Then they were washed, and placed in an acid-alcohol solution for 10 s. Subsequently, a series of three washes using distilled water were conducted, followed by immersion in Biebrich's scarlet acid fuchsin solution for 10 min, 1 % phosphomolybdic acid solution for 2 min, aniline blue solution for 10 min, and acetic acid solution for 1 min. After dehydration in graded alcohol, the slices were sealed and inspected under a microscope (Nikon, Japan).

### 2.8 TUNEL staining

The spleen tissue slides were permeated in 0.1 % Triton for 5 min at room temperature. Then, the spleen sections were blocked with 5 % BSA for 30 min and then washed with PBS for 3 times. Next, the spleen sections were incubated with TUNEL reagent mixture. After 1 hour of incubation, the cell nucleus was counterstained with DAPI, and the slides were sealed and photographed under a microscope (Nikon, Japan).

### 2.9 Electron microscope analysis

The spleen of severely diabetic mice was cut into a volume of 1 mm × 1 mm × 1 mm and fixed in pre-cooled 3 % glutaraldehyde (containing 2 % paraformaldehyde) at 4 °C overnight. The tissue was placed on the wax plate with fixative, and cut into slices with a sample thickness of 100 nm. After soaking in the fixative, the slices were fixed on a copper grid, stained with 0.2 % uranyl acetate solution, and then the slices were observed using a 120 KV transmission electron microscope (TEM) (Tecnai G2 SPIRIT, FEI Corporation, USA). The structure and elemental composition of endogenous particles in unstained spleen tissue samples were characterized using high-angle annular dark field scanning transmission electron microscopy (HAADF-STEM) (Titan Themis Z microscope, Thermo Fischer Scientific, USA) with elemental mapping analysis of energy-dispersive X-ray spectroscopy (EDS), operated at 300 kV.

### 2.10 Statistical analysis

Data were presented as mean ± standard deviation (SD) and analysed using the SquidLab program to fit SQUID data [24]. Statistical analysis was conducted using GraphPad Prism 9.0 (GraphPad Software, CA, USA), For magnetic susceptibility values, the data between two groups were analyzed by the unpaired Student's *t*-test. Differences between groups were considered significant when *P* < 0.05. We used Image J 1.0 and Origin 2019 for data analysis.

## 3 Results

### 3.1 Mouse organs are overall diamagnetic, but contain paramagnetic components, especially in the spleen

**Fig. 1:**
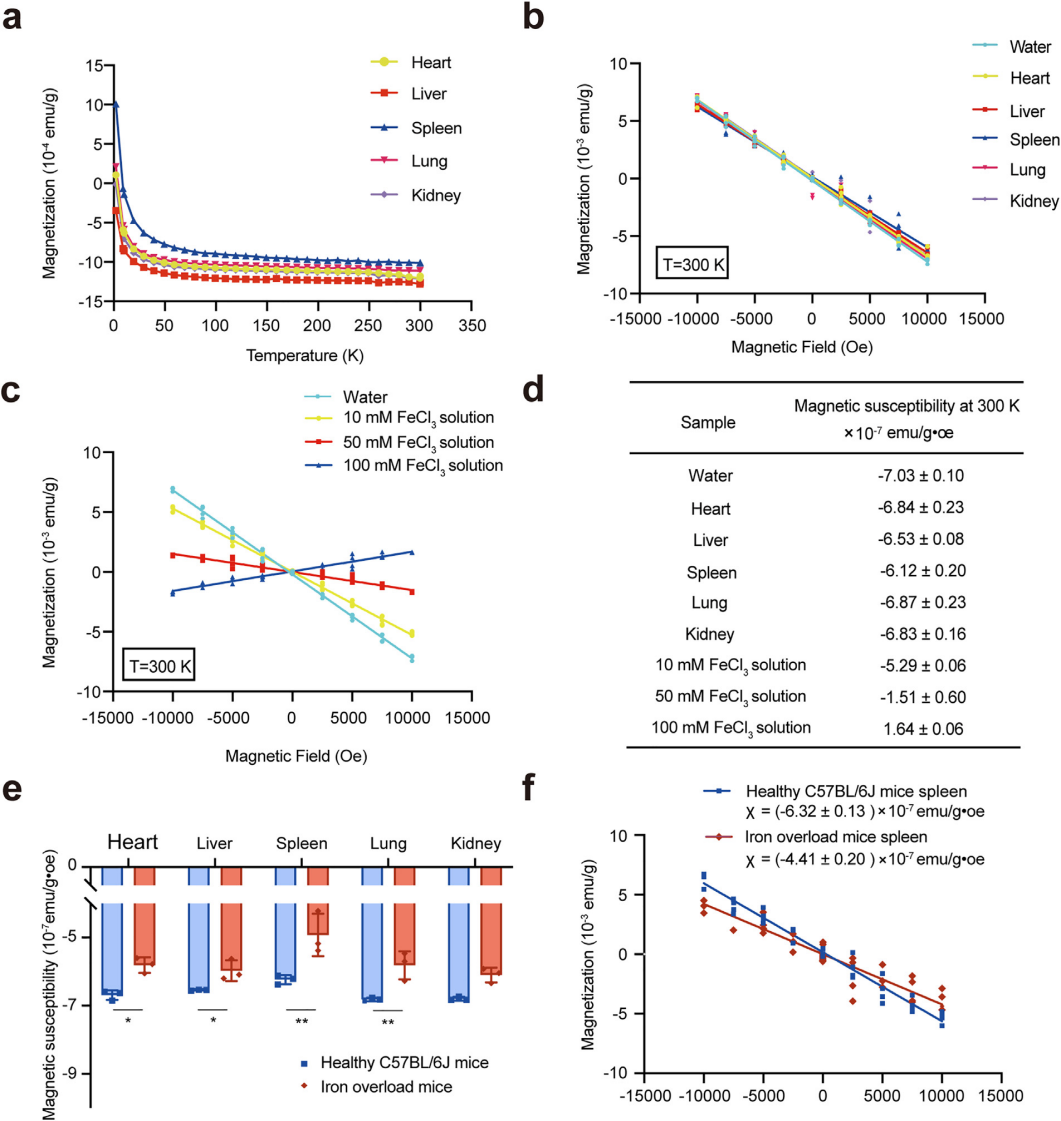
**Mouse organs are overall diamagnetic, but their iron contents directly affect their magnetic properties.** Measurement performed by a SQUID MPMS3. (a) Representative M-T (magnetization-temperature) curves of heart, liver, spleen, lung and kidney tissues of healthy C57BL/6J mice. (b) Representative M-H (magnetization-magnetic field strength) curves of heart, liver, spleen, lung and kidney tissues of healthy mice at 300 K. (c) Representative M-H curves of water and FeCl_3_ solution with three different FeCl_3_ concentrations, 10 mM, 50 mM and 100 mM. (d) Calculated magnetic susceptibilities of samples tested in (b, c). (e) Magnetic susceptibilities of major organs of healthy C57BL/6J mice vs. C57BL/6J mice supplemented with excess iron were measured by SQUID at 300 K and calculated. Data represent mean ± SD (*n* = 3 mice per group). * *P* < 0.05, ** *P* < 0.01. (f) Representative M-H curves of the spleen tissue in a healthy C57BL/6 J mice vs. a C57BL/6 J mice supplemented with excess iron. Four data points were collected under each non-zero magnetic field (six data points under zero field), and M-H curves were obtained after fitting. Measurements were performed at 300 K by SQUID.

We first investigated the magnetic properties of heart, liver, spleen, lung, and kidney tissue samples obtained from healthy C57BL/6 J mice using a MPMS SQUID magnetometer. We used ceramic tools to collect fresh tissues from the mice to avoid possible iron contamination, and the blood was also excluded. Tissue samples were collected and immediately placed on ice before being measured within 3 h. By analyzing the magnetization versus temperature (M-T) curves, we found that the magnetization of all tissue samples showed an increase at low-temperature, indicating the presence of paramagnetic components, especially the spleen (Fig. 1a).

To get the precise magnetic properties of different tissues, we measured the organ M-H curve at 300 K to mimic physiological conditions, and also compared it with double distilled water. The results reveal a negative correlation of the magnetization of all samples with the magnetic field strength (Fig. 1b), confirming their overall diamagnetism, which is similar to the distilled water. This is not surprising because it is known that the organs have 60 %−70 % of water. In fact, we measured the magnetic properties of 10 mM, 50 mM and 100 mM FeCl_3_ solutions and found that only the 100 mM FeCl_3_ solution exhibited overall paramagnetism (1.51 × 10^–7^ emu/g•oe), while both 10 mM and 50 mM FeCl_3_ solutions exhibited overall diamagnetism (Fig. 1c). This confirms that only when the paramagnetic components in a diamagnetic background reach a threshold, the overall magnetic susceptibility could apear to be paramagnetic. Moreover, the amount of paramagnetic components in the solution are directly correlated with the overall magnetic susceptibility (Fig. 1c,d). The 50 mM FeCl_3_ solution has a magnetic susceptibility of −1.51 × 10^–7^ emu/g • oe, which is much less diamagnetic than 10 mM FeCl_3_ solution whose magnetic susceptibility is −5.29 × 10^–7^ emu/g•oe, because 50 mM FeCl_3_ solution has 5-fold of paramagnetic components than 10 mM FeCl_3_ solution.

### 3.2 Iron supplement affects organ tissue magnetic susceptibility

Our results show that the average mass magnetic susceptibility of the mouse organ samples ranges from −6.24 × 10^–7^ to −6.82 × 10^–7^ emu/g•oe, which are less diamagnetic than water (−7.03 × 10^–7^ emu/g•oe) (Table S1). Among them, spleen tissue, the largest iron storage organ, displays the most significant difference (−6.12 × 10^–7^ emu/g•oe) (Fig. 1d). In fact, DAB-enhanced Perls staining also confirms that spleen tissue has a higher level of iron than heart, liver, lung and kidney tissues (Fig. S1).

To investigate the effects of iron content and organ magnetic properties, we administered ferrous sulfate to the C57BL/6 J mice by gavage for a duration of 10 weeks. We collected their heart, liver, spleen, lungs, and kidneys and measured their mass magnetic susceptibility (Table S2). There is a general increase in the magnetic susceptibilities for all the organs in the iron-overload mice (Fig. 1e), which is consistent with the contribution of paramagnetic iron. Among them, the changes of the spleen and lung tissues are the most significant. Specifically, the average mass magnetic susceptibility of the spleen in iron-overload mice is −4.92 ± 0.6 × 10^–7^ emu/g•oe, while that of the healthy C57BL/6 mice is −6.24 ± 0.13 × 10^–7^ emu/g•oe. The average mass magnetic susceptibility of the lung in iron-overload mice is −5.81 ± 0.6 × 10^–7^ emu/g•oe, while that of the healthy C57BL/6 mice is −6.82 ± 0.05 × 10^–7^ emu/g•oe. The iron-overload has reduced their diamagnetism by 21 % and 14.7 %, respectively. However, it should be pointed out that the overall organ still appears to be diamagnetic (Fig. 1f) because the major contribution to the overall magnetic susceptibilities still comes from diamagnetic water in the organs.

### 3.3 Magnetic susceptibility changes in the spleen tissue of diabetic mice

**Fig. 2:**
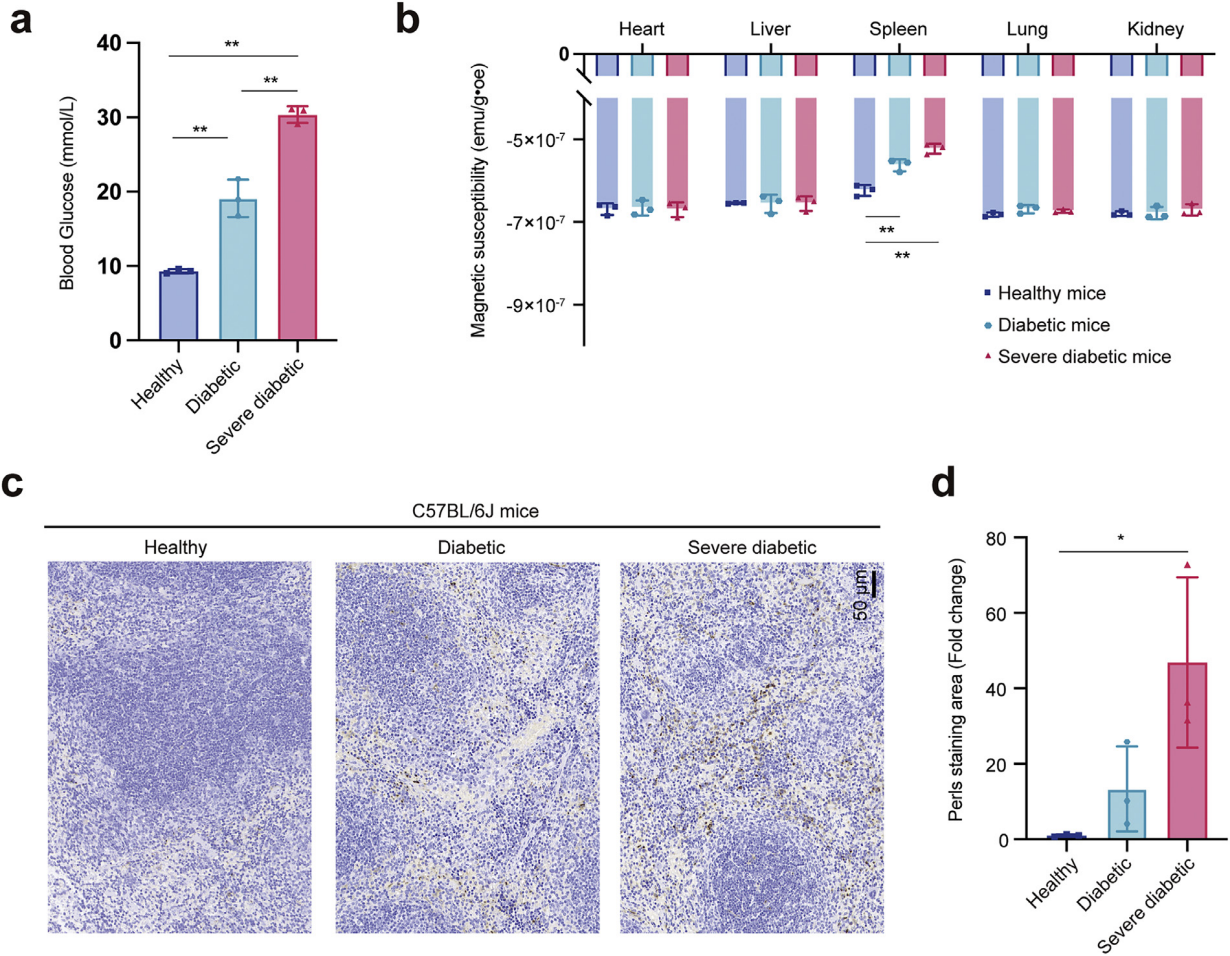
**Diabetic mouse spleen has obviously changed magnetic susceptibility and iron content.** (a) Blood glucose levels of healthy C57BL/6J mice vs. two different types of diabetic mice. (b) Magnetic susceptibilities of major organs of healthy C57BL/6J mice vs. two different diabetic mice. Measurements were performed at 300 K by SQUID. (c) DAB-enhanced Perls staining of the spleen tissues of healthy C57BL/6J mice vs. two different types of diabetic mice. Scale bar: 50 µm. (d) Quantification of the DAB-enhanced Perls staining area in the spleen tissues of healthy C57BL/6J mice vs. two different types of diabetic mice. Data represent mean ± SD (*n* = 3 mice per group). * *P* < 0.05, ** *P* < 0.01.

The general iron-overload caused by iron supplements does not occur that frequently, but iron metabolism abnormalities are very common in multiple pathological conditions, including diabetes. To investigate the magnetism of organ tissues in healthy vs. diabetic mice, we used different doses of STZ, which impairs the pancreatic cell functions, to generate two different diabetic mouse models (“regular” vs. “severe”). For the “regular” diabetic mice we used, the blood sugar level is ∼20 mM, while for the “severe” diabetic mice, the blood sugar level is ∼30 mM, which are both significantly higher than the healthy control mice of ∼10 mM (Fig. 2a). Our results show that the magnetic susceptibilities of the spleen tissues are significantly altered in different types of mice, but not in the heart, liver, lung or kidney (Fig. 2b; Table S3). The decreased diamagnetism of the spleen of diabetic mice indicates a higher content of paramagnetic substances. To examine whether the iron content changes in these mice, we used DAB-enhanced Perls staining, which exhibited a substantial augmentation in the quantity of iron deposition in diabetic mice, especially in the severe diabetic mice (Fig. 2c). We counted the positive areas of DAB-enhanced Perls staining and found a progressive increasing trend in the content of iron deposition correlated with the disease severity in the spleen (Fig. 2d), but not in the liver (Fig. S2).

### 3.4 Gradient high magnetic field damages spleen tissue of diabetic and iron-overload mice

**Fig. 3:**
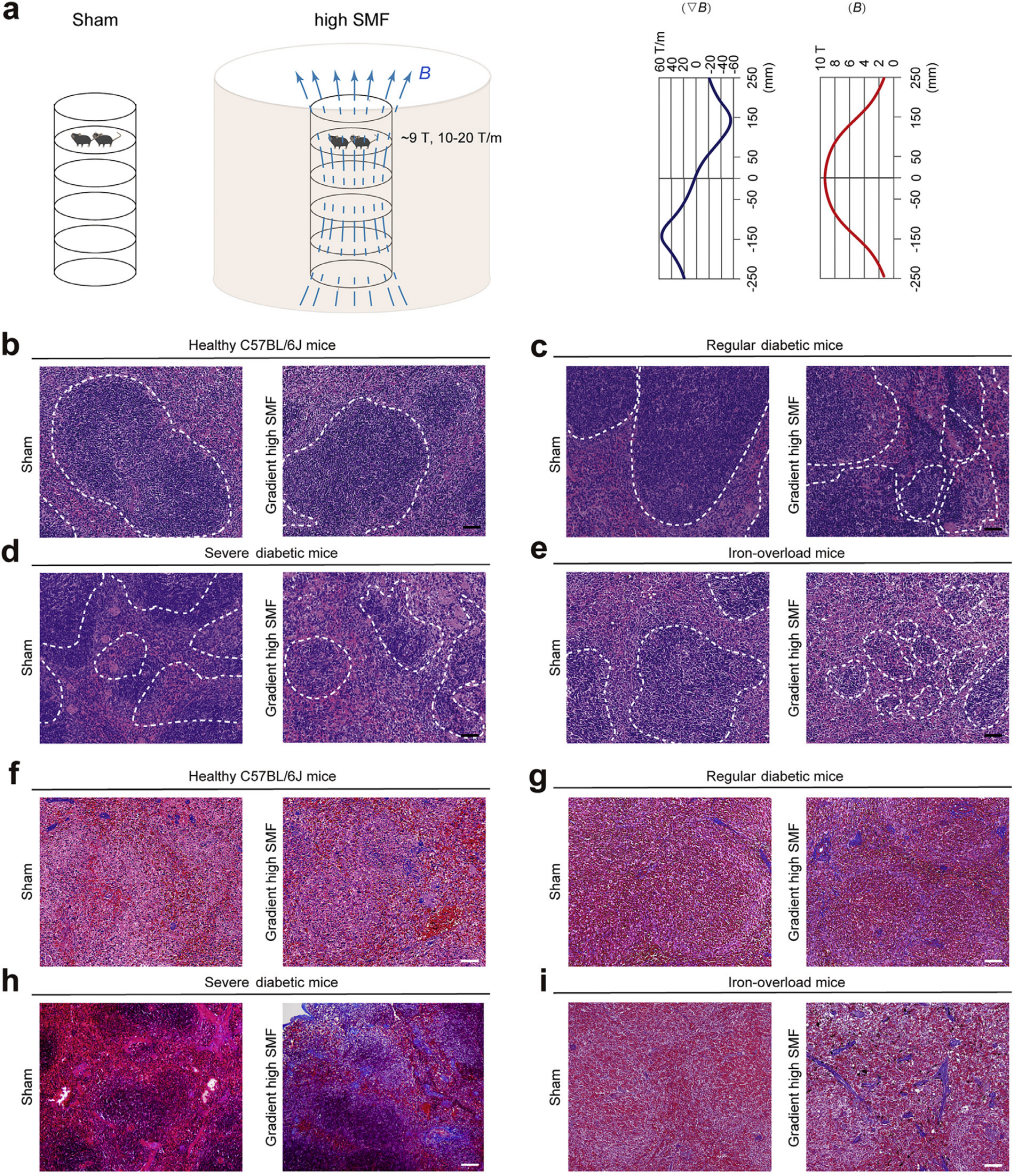
**Gradient high magnetic field damages iron-overloaded mouse spleen.** (a) Illustration of the experimental set up in the superconducting magnet. The mice were exposed to (10–20 T/m, 9 T gradient high SMF for 14 h. HE staining of sham or gradient high SMF-treated (b) healthy C57BL/6 J mice, (c) regular diabetic mice with blood glucose of ∼20 mM, (d) severe diabetic mice with blood glucose of ∼30 mM and (e) iron-overload mice. The outlined purple areas are while pulp of the spleen. The surrounding areas are red pulp of the spleen, where the blood is filtered and red blood cells are recycled. Masson's trichrome staining of sham or gradient high SMF-treated (f) healthy C57BL/6 J mice, (g) regular diabetic mice with blood glucose of ∼20 mM, (h) severe diabetic mice with blood glucose of ∼30 mM and (i) iron-overload mice. Scale bar: 50 µm.

Our previous research shows that gradient high magnetic field treatment for 14 h can increase the lethality of severe diabetic mice [25]. To elucidate the potential impact of tissue magnetic susceptibility on the biological responses within the magnetic field, we exposed healthy C57BL/6 J mice, regular diabetic mice (blood sugar level is ∼20 mM), severe diabetic mice (blood sugar level is ∼30 mM) and iron overloaded mice to a ∼9 T SMF with a gradient (∇B) of 10–20 T/m for 14 h, placed away from the magnet center (Fig. 3a). Hematoxylin-eosin (HE) staining results show that this prolonged treatment of gradient high magnetic field did not generate obvious spleen impairment in the healthy C57BL/6 J mice (Fig. 3b), and only induced slight damage to the spleen of regular diabetic mice (Fig. 3c). In contrast, prolonged treatment of gradient high magnetic field induced evident spleen tissue damage in the severe diabetic mice (Fig. 3d) as well as the iron-overload mice (Fig. 3e), which have reduced lymphoid follicle size and spleen lymphocyte count compared with the sham group. Moreover, we also used Masson's trichrome staining to examine the fibrosis in the mice spleen, which also confirms the differential effects of gradient high magnetic field on different mice (Fig. 3f-i).

**Fig. 4:**
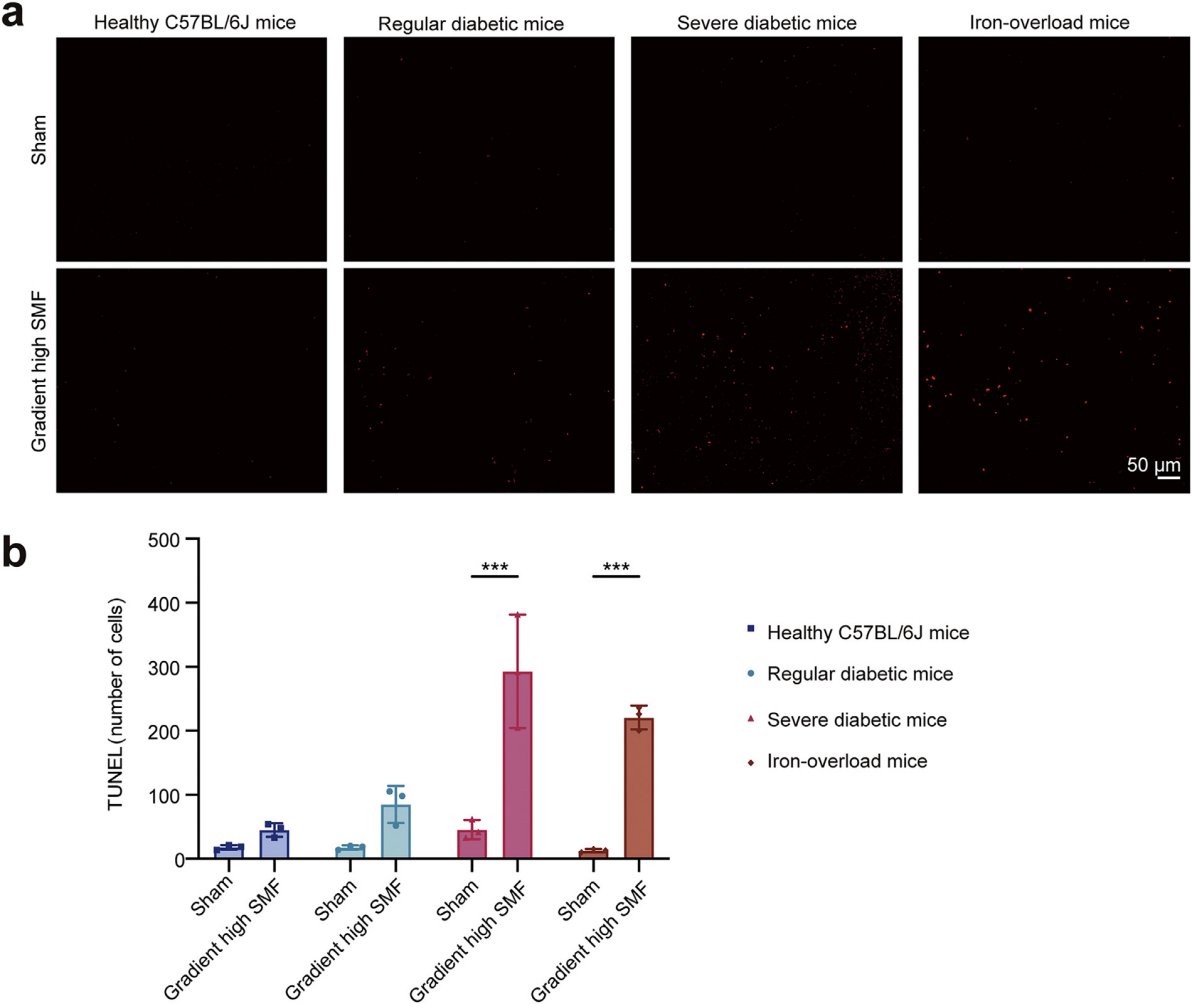
**Gradient high magnetic field increase apoptosis level of the spleen.** (a) Representative images and (b) quantifications of TUNEL-staining of sham or gradient high SMF-treated healthy C57BL/6 J mice, regular diabetic mice with blood glucose of ∼20 mM, severe diabetic mice with blood glucose of ∼30 mM and iron-overload mice spleen. Scale bar: 50 µm. Data represent mean ± SD (*n* = 3 mice per group). * *P* < 0.05, ** *P* < 0.01.

To further examine the spleen damage at cellular levels, we also performed TUNEL staining, which stains for apoptotic cells (Fig. 4a). Our results show that there was no significant difference in the apoptosis level of the spleen between healthy mice and regular diabetic mice after high-gradient magnetic field treatment. However, the apoptosis levels of the spleen tissue of severe diabetic mice and iron-overloaded mice were significantly increased (Figs. 4b and S3).

### 3.5 Iron accumulation in the red pulp of the diseased mice spleen leads to endogenous iron biomineralization

**Fig. 5:**
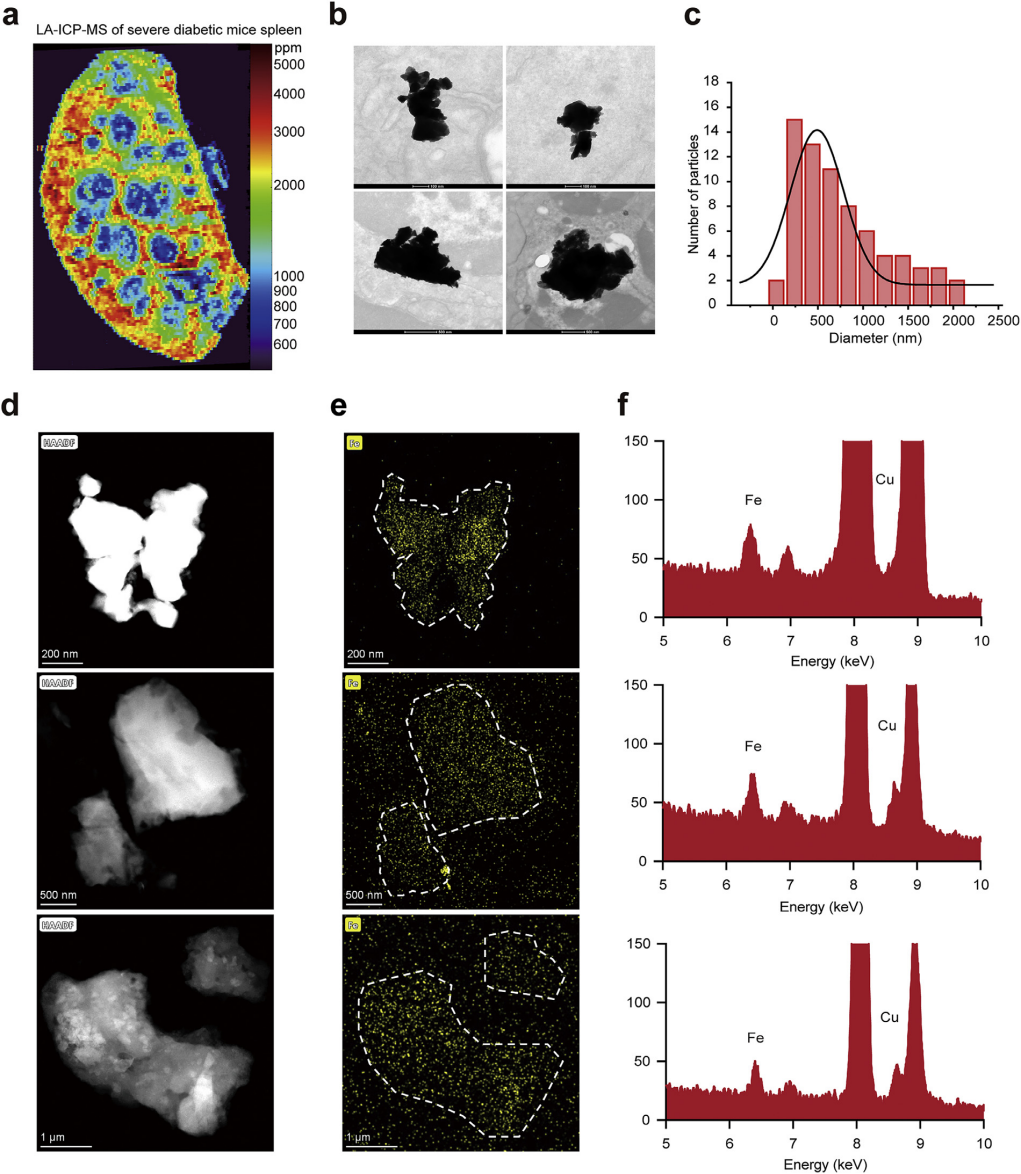
**Iron accumulation leads to endogenous particle formation in the red pulp of severe diabetic mouse spleen.** (a) LA-ICP-MS element mapping confirm that iron deposition specifically occurs in the spleen red pulp. (b) Representative TEM images of iron accumulation in the red pulp of the severe diabetic mouse spleen forms endogenous particles. Scale bar: 100 or 500 nm. (c) Particle size distribution of the endogenous particles from the TEM pictures. (d) Representative HAADF-STEM image of particle aggregates from tissue samples from severe diabetic mice. Scale bar: 200 nm to 1 µm. (e) The EDS mapping of the particle aggregates confirm the presence of Fe. Scale bar: 200 nm to 1 µm. (f) The EDS spectrum further confirming the signal from the element Fe.

In the results of DAB-enhanced Perls staining of mouse spleen tissue in Fig. 2c, we noticed that the iron is specifically enriched at the red pulp area in the spleen, where the blood is filtered and old/damaged red blood cells are recycled. In fact, LA-ICP-MS element mapping was used to examine the severe diabetic mice, which clearly demonstrates that iron deposition obviously and specifically occurs in the spleen red pulp, but not the white pulp (Fig. 5a).

To get a closer look at the iron deposition in the mouse spleen, we performed TEM of the spleen tissue of severe diabetic mice, which unraveled the formation of large particles inside the spleen (Figs. 5b and S4). These particles range from 124.4 nm to 1 µm, with an averaged diameter of 780.8 ± 516.1 nm (Fig. 5c), which is unlikely through externally inhaled pathways. To further validate their composition, we performed HAADF-STEM combined with EDS and confirmed that these endogenous particles all contain Fe elements (Fig. 5d-f). This indicates that these iron biomineralizations could contribute to the increased magnetic susceptibilities, especially at the red pulp region.

**Fig. 6:**
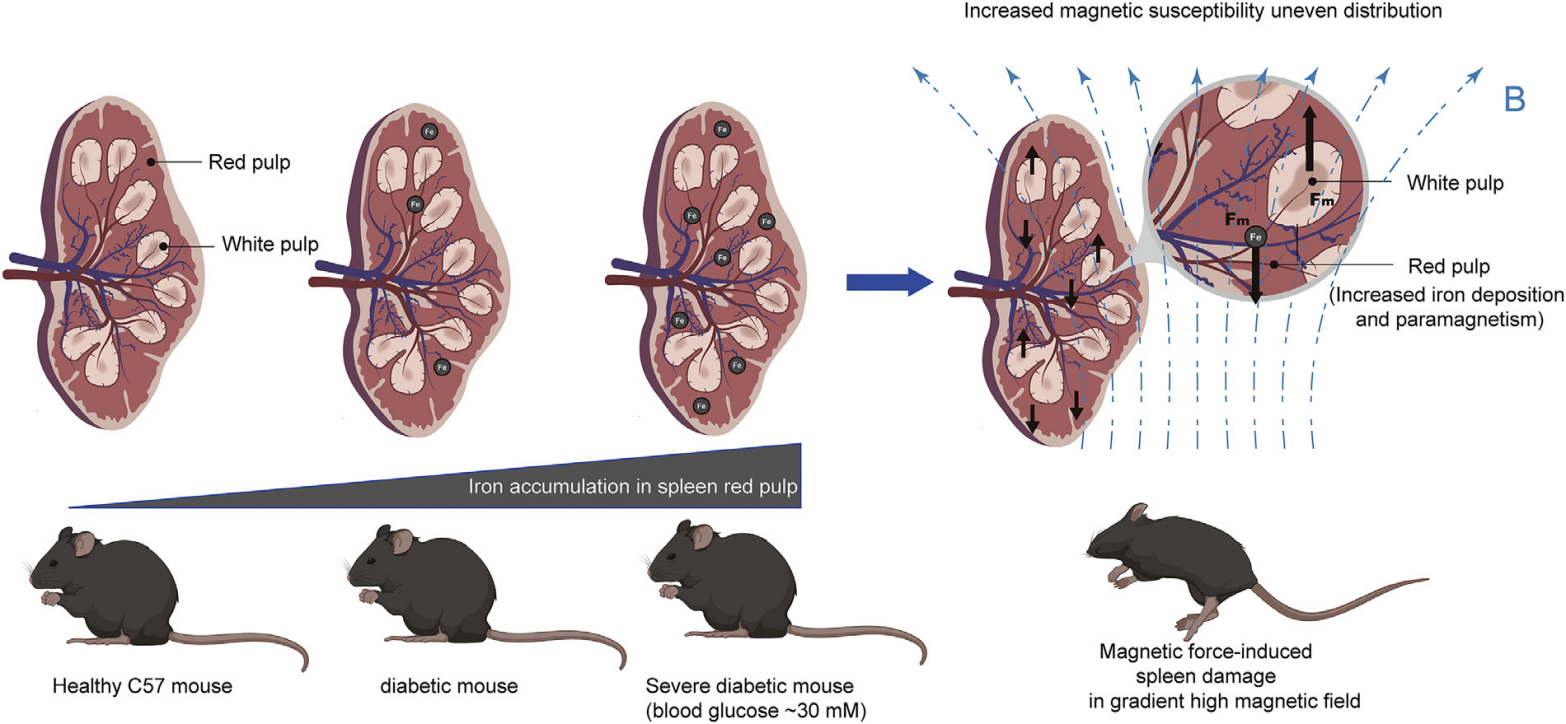
**Iron accumulation-induced endogenous particle formation in spleen red pulp damages spleen of severe diabetic mice in gradient high magnetic field**.

Since magnetic forces are proportional to the magnetic flux density (B), magnetic field gradient (∇B) and magnetic susceptibilities of the object, diamagnetic object and paramagnetic object will experience oppsite magnetic forces in gradient field. The uneven distribution of the magnetic susceptibilities inside the spleen leads to uneven magnetic forces when they are placed in gradient high magnetic fields (Fig. 6). More specifically, under gradient high magnetic field, the iron biomineralization in the red pulp will experience opposite magnetic forces compared with the white pulp, which has low level of iron and will be more diamagnetic. Our results show that when diabetic condition gets more severe, the iron accumulation get more evident, which leads to iron biomineralization in red pulp, uneven magnetic susceptibilities and magnetic forces inside the spleen. Consequently, prolonged gradient high magnetic field treatment generates spleen damage in these severe diabetic mice.

## 4 Discussion

Magnetic property of matter is the major determining factor for their response to external magnetic fields, which is also the basis for detection techniques including magnetic resonance imaging (MRI). For human bodies, it is known that the organ magnetic susceptibilities are close to water because water constitutes 60 %−70 % of most living organisms. This is also the reason why a frog can be magnetically levitated in a gradient high magnetic field provided by a superconducting magnet. In fact, this is also why human bodies can be safely examined using high-field MRI, which is ∼100,000 fold higher than the geomagnetic field. However, our research confirms that tissues exhibit distinct magnetic properties under some pathological conditions. Notably, a significant difference in magnetic susceptibility values was observed between spleen tissues of diabetic vs. healthy mice, which is associated with increased iron content. Additionally, in experiments measuring safety and biological effects, severe damage was observed in the spleens of severe diabetic mice as well as mice with excess iron supplement under a gradient high static magnetic field. These findings will have important implications for the safe application of high magnetic field-related techniques on individuals with abnormal iron metabolism.

Iron metabolism abnormality is frequently associated with the progressive accumulation of iron in various tissues. Enhanced iron deposition can induce oxidative stress and endoplasmic reticulum stress, which damage cellular lipids, proteins, and nucleic acids, ultimately leading to organelle damage and dysfunction [26,27]. The toxic effects of iron overload include cell apoptosis or necrosis in organs such as the heart, pancreas, and spleen, as well as hemosiderin deposition in various tissues [28]. Iron overload can have a devastating effect on pancreatic β cells, and is often seen in patients with β-thalassemia who are frequently transfused [29]. Another iron overload disease that affects β cells is hereditary hemochromatosis. Patients are unable to respond to increased insulin secretion because of damage to β cells [18]. Other studies have shown that diabetic rats supplemented with iron have greater oxidative injury in the pancreas and heart, which is related to iron accumulation in tissue [30]. In diabetic nephropathy, iron deposition occurs in kidney tissue and can induce renal fibrosis [31]. Moreover, it has been shown that iron chelators can effectively reduce tissue damage in metabolic diseases [32,33].

In our study, we demonstrate that the iron metabolism dysregulation can lead to high levels of iron accumulation, which form larger-sized iron particles in the red pulp of spleen. It has been shown that bacteria can form mineralized precipitated iron minerals through iron biomineralization after culture in solutions containing iron [34,35]. People have also found iron crystallization in two postmortem spleen tissues with hemosiderinosis and hereditary spherocytosis [36], but the correlation between high levels of iron levels and endogenous biomineralization has not been studied. Since iron is an essential component of the hemoglobin molecules in red blood cells, and the spleen red pulp is the location where the blood is filtered and red blood cells are recycled, it is not surprizing that iron accumulation occurs more evidently in the spleen red pulp compared with other tissues. Besides, the increased iron accumulation in the red pulp area of the diseased mice spleens are also likely originated from macrophages, which can precipitate and store iron deposits [37]. In fact, macrophages were also identified to be the reason why magnetic particles have been found in the beak of the pigeon [9].

Our study demonstrated the endogenous origin of these iron-containing particles in diseased mice. Since our mice were SPF (specific pathogen free) graded and were housed in the barrier animal facility throughout their life, which excludes the possibility of exogenously inhaling iron contamination. We also used ceramic and plastic tools, instead of metal, throughout the whole experiment to avoid any possible contamination from the experimental procedure. Moreover, the size of these particles is around 800 nm, which is too large to be inhaled and filtered through airway and blood stream. Furthermore, although it has been shown that red pulp macrophages are superparamagnetic [38], we only observed particle formation in the severe diseased mouse spleen, but not in the healthy mice's spleen or the other major organs we examined. However, we are aware that these particles are quite different from magnetite in magnetotactic bacteria. Besides iron, there are also other elements, such as calcium, included in these particles during biomineralization, and these particles are not really crystals. We hypothesize that this biomineralization may also occur in other iron metabolism disorder related diseases, such as Alzheimer's and Parkinson's diseases, which also have aberrant iron metabolism and may lead to potential iron particle formation in the brain. Although these particles are not likely be responsible for geomagnetic sensing, they could lead to altered magnetic properties in diseased conditions, which can be used in diagnosis in the future.

Although water is the main component of most tissues and exhibits diamagnetism [39], we found differences in mass magnetic susceptibility between tissues and water, with spleen tissue exhibiting the most significant change (Fig. S5). Using FeCl_3_ solutions with different concentrations, we show that the magnetic susceptibility is directly related to the amount of paramagnetic content in the water. For lower concentration of 10 mM FeCl_3_, the magnetic susceptibility is −5.29 × 10^–7^ emu/g·oe, which appears to be diamagnetic as a whole. However, when we increase the FeCl_3_ concentration to 100 mM, the magnetic susceptibility becomes 1.64 × 10^–7^ emu/g·oe, which is apparently paramagnetic. The high content of diamagnetic water in biological systems masks the paramagnetic and even ferromagnetic substances in our bodies. In fact, the biological systems also contain many other components that can make the magnetic properties even more complicated. For example, the magnetic susceptibility of paramagnetic hemoglobin systems is affected by metal ions and porphyrins [40]. Therefore, in the diamagnetic background of biological systems, the existence of paramagnetic and/or ferromagnetic substances have a great potential to be developed as diagnostic indicators.

## 5 Conclusion

We not only demonstrated the endogenous iron biomineralization formation in metabolic diseased mice, but also revealed a direct link among the iron content, tissue magnetic susceptibility and magnetic field bioeffects. Although the biological systems appear diamagnetic as a whole, the increased iron content changes their magnetic susceptibility. Iron metabolism dysregulation in diseases such as severe diabetes leads to iron accumulation and endogenous iron biomineralization in the red pulp of the spleen, which filters blood and degrades red blood cells. Consequently, the magnetic susceptibility differences between red pulp and white pulp are increased, which caused spleen damage in gradient high magnetic field due to the magnetic forces. It is possible that this iron accumulation-induced iron biomineralization and magnetic field sensitivity changes can also occur in other iron metabolism dysregulation-related diseases such as Alzheimer's and Parkinson's diseases.

## For studies with animals

The study was conducted according to the National Institutes of Health Guide for the Care and Use of Laboratory Animals (NIH publication, 8th Edition, 2011). Moreover, the procedures were approved by the Biomedical Ethics Committee of Anhui Medical University (LLSC20232243).

## CRediT authorship contribution statement

X.Z. and R.G. designed the experiments. R.G., L.Z., D.S., B. Y., C.S., H.C., W.X., C.F., G.C., Z. Q., and H.D. performed the experiments and analysis. X.Z. and R. G. wrote the manuscript with editing by all authors.

## Declaration of competing interest

The authors declare that they have no conflicts of interest in this work.

## Data Availability

All the data needed to evaluate the conclusions in the paper are present in the paper and/or the Supplementary information.

## Appendix Supplementary materials

Image, application 1
