# Supplementary material for: Endogenous iron biomineralization in the mouse spleen of metabolic diseases
Source: Fundam Res. 2024 Jul 25;6(3):1535–44. doi: 10.1016/j.fmre.2024.07.004 (PMC13247509; doi:10.1016/j.fmre.2024.07.004)
Supplement: Supplementary file 1 [file mmc1.docx]

Supplementary Information

**
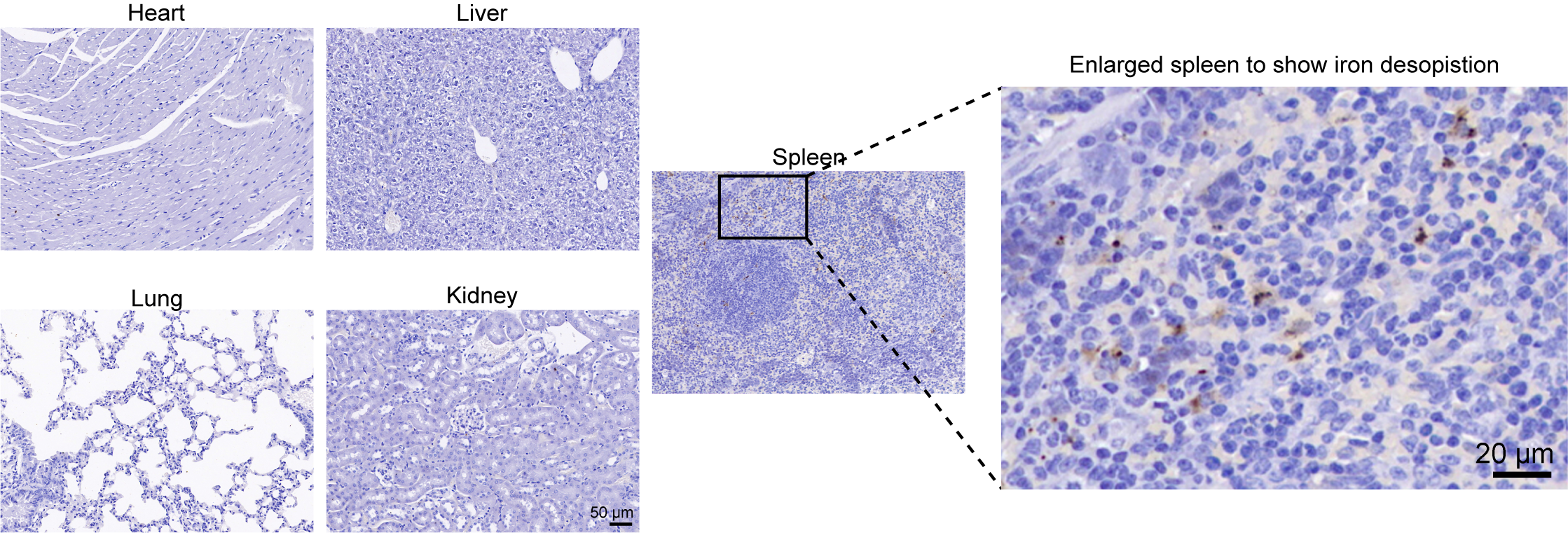
Fig. S1. DAB-enhanced Perls staining of heart, liver, lung, kidney and spleen tissues of healthy C57BL/6J mice.** Scale bar: 20 or 50 μm.

**
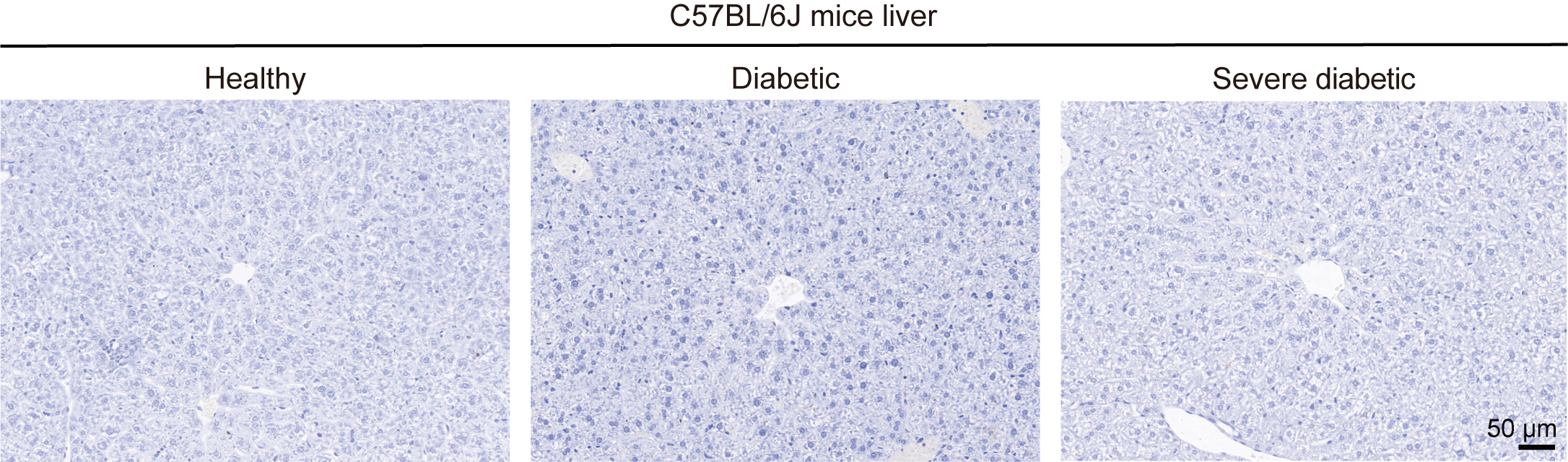
**

**Fig. S2.** **No obvious iron deposition in the mice liver.** The iron content in the liver tissues of the three groups of mice was detected by DAB-enhanced Perls staining. Scale bar: 50 μm.


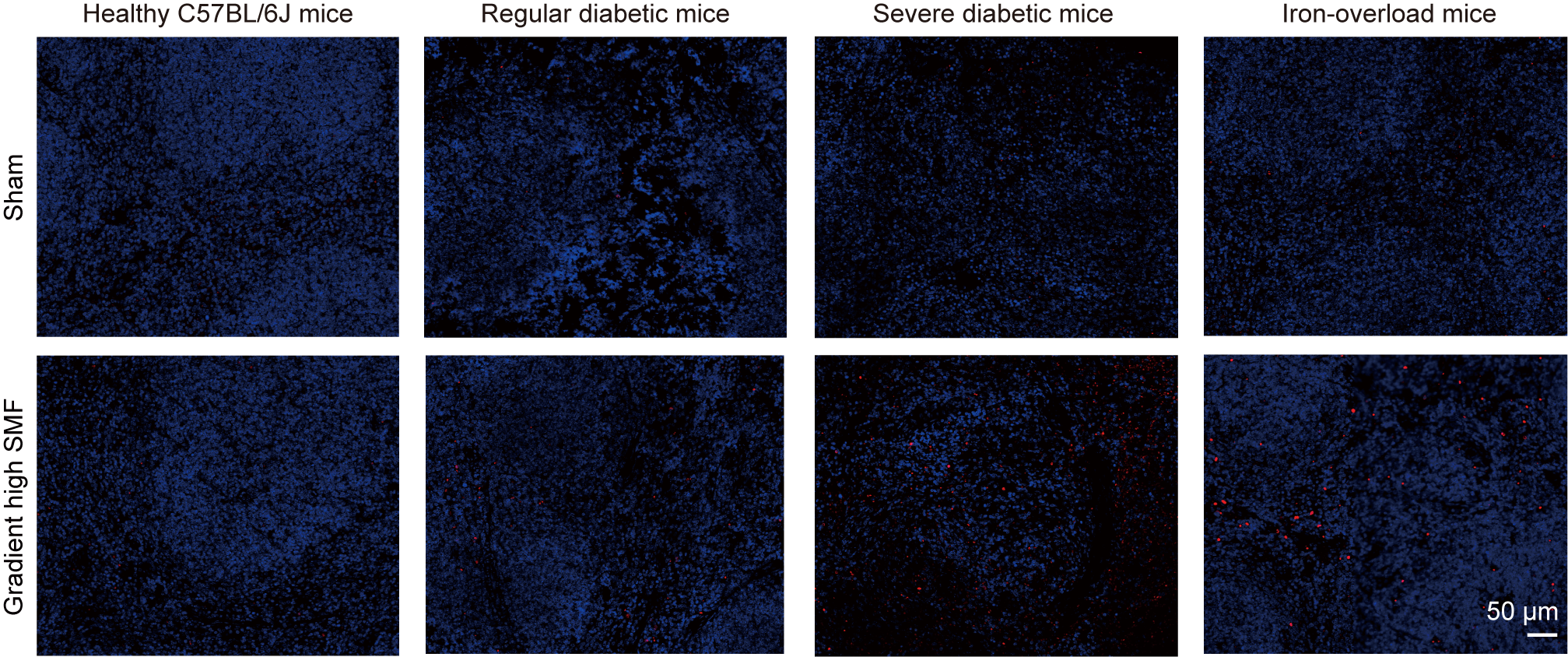


**Fig.S3. Gradient high magnetic field increase apoptosis levels in mice spleen with iron accumulation.** TUNEL-staining of sham or gradient high SMF-treated healthy C57BL/6J mice, regular diabetic mice with blood glucose of ~20 mM, severe diabetic mice with blood glucose of ~30 mM and iron-overload mice spleen. Blue, DAPI; Red, TUNEL. Scale bar: 50 μm.


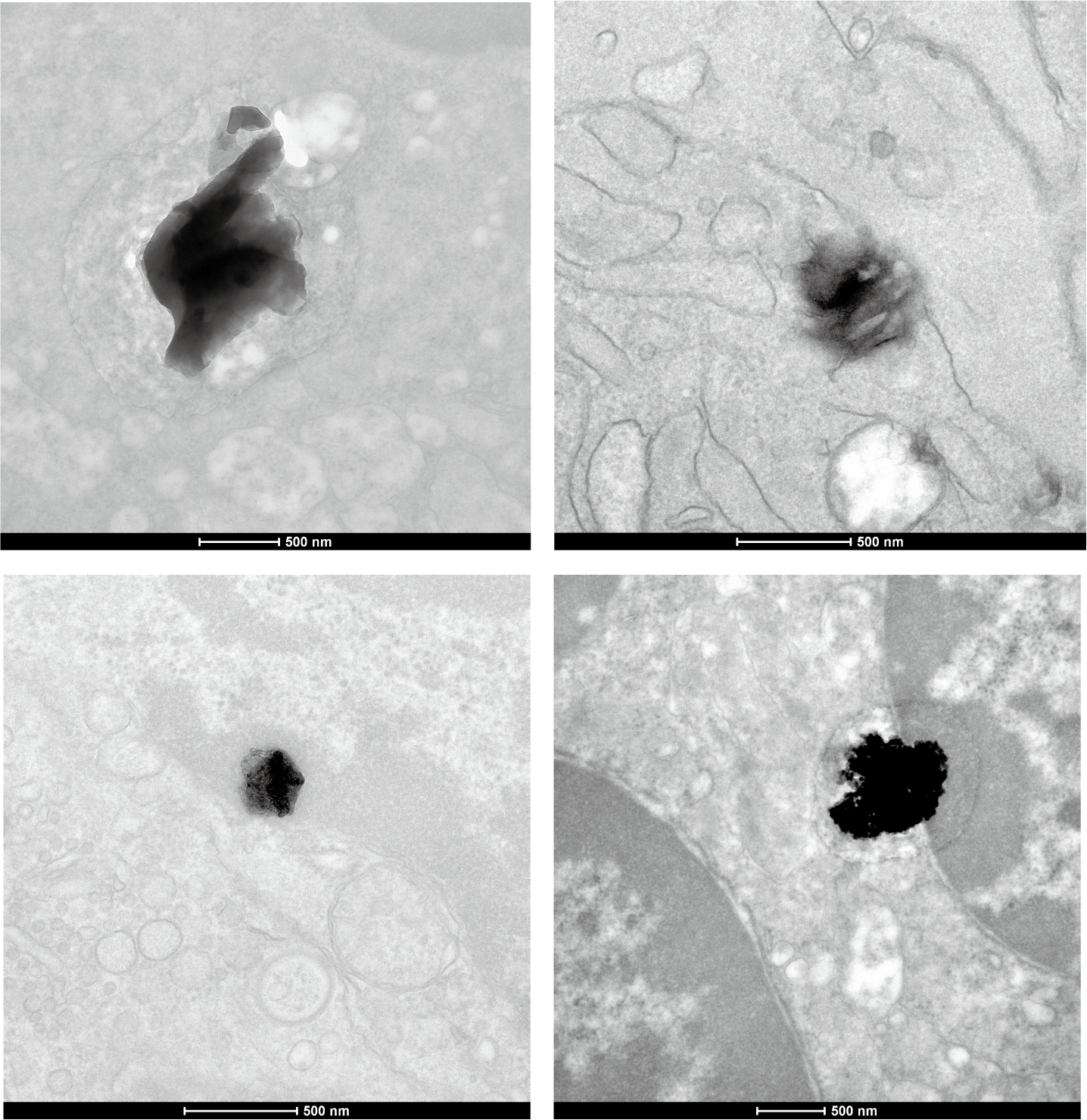


**Fig. S4. Iron deposition in spleen tissues of diabetic mice.** Scale bar: 500 nm.


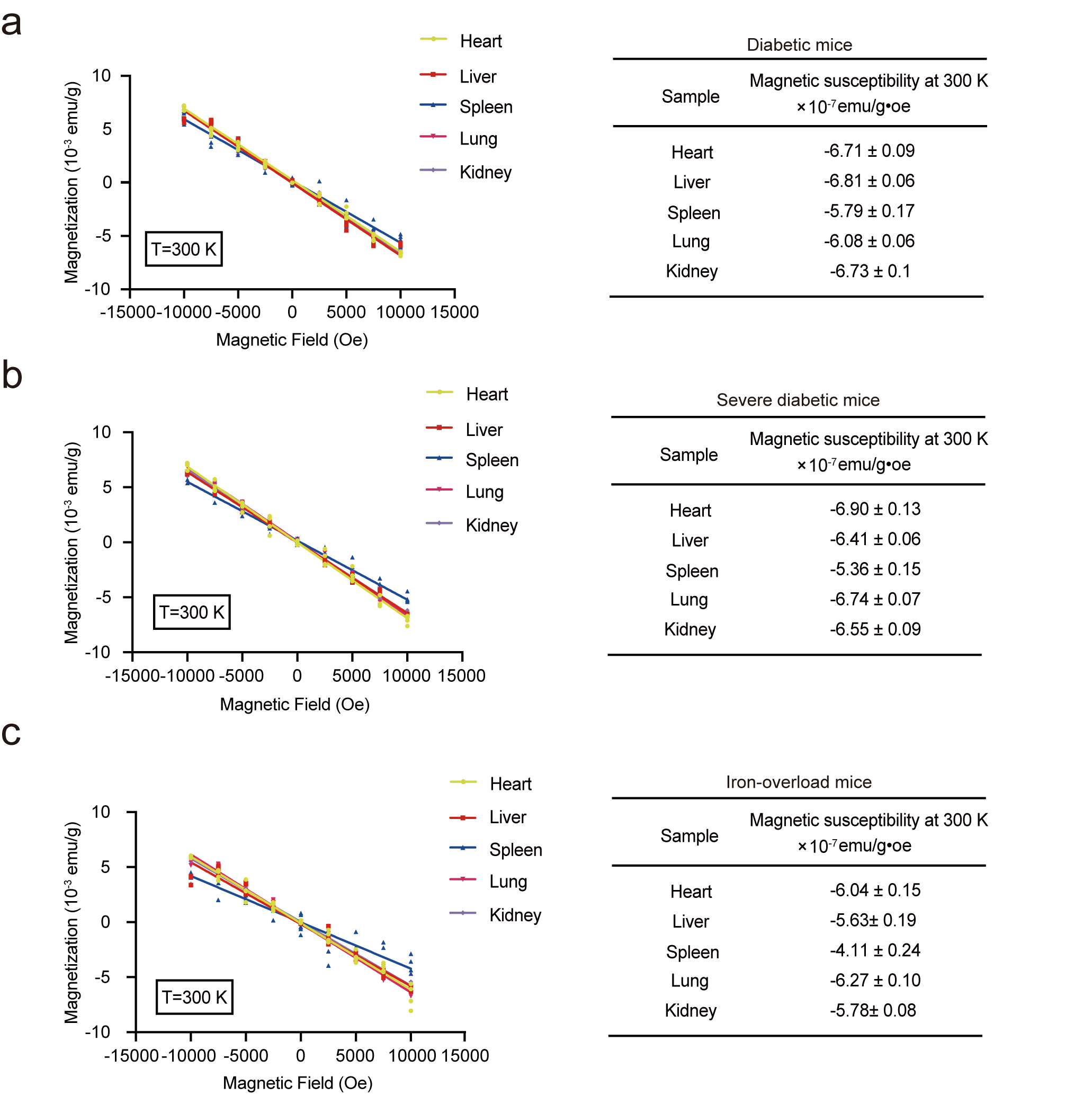


**Fig. S5. Metabolic diseased mice organs are diamagnetic.** M-H curves and magnetic susceptibilities of heart, liver, spleen, lung and kidney tissues in diabetic mice (a), severe diabetic mice (b), iron overload mice (c). Measurements were performed at 300 K by SQUID.

**Table S1. Mass magnetic susceptibility χ_mass_ of C57BL/6J mice tissues measured at 300 K.**

| **Sample** | **Mass magnetic susceptibility χ_mass_, (× 10^-7^ emu/g** • **oe)** |
| --- | --- |
| Heart | -6.69 ± 0.10 |
| Liver | -6.54 ± 0.01 |
| Spleen | -6.24 ± 0.13 |
| Lung | -6.82 ± 0.05 |
| Kidney | -6.80 ± 0.06 |
| Double distilled Water | -7.03 ± 0.10 |

**Table S2. Mass magnetic susceptibility χ_mass_ of iron supplement mice tissues measured at 300 K.**

| **Animal** | **Sample** | **Mass magnetic susceptibility χ_mass,_ (× 10^-7^ emu/g** • **oe)** |
| --- | --- | --- |
| Iron-overload mice | Heart | -5.81 ± 0.1 |
|  | Liver | -5.97 ± 0.2 |
|  | Spleen | -4.92 ± 0.6 |
|  | Lung | -5.81 ± 0.4 |
|  | Kidney | -6.10 ± 0.2 |

**Table S3. Mass magnetic susceptibility χ_mass_ of diabetic mice tissues at 300 K.**

| **Animal** | **Sample** | **Mass magnetic susceptibility χ_mass,_ (× 10^-7^ emu/g • oe)** |
| --- | --- | --- |
| T1DM mice | Heart | -6.66 ± 0.10 |
|  | Liver | -6.56 ± 0.20 |
|  | Spleen | -5.62 ± 0.10 |
|  | Lung | -6.69 ± 0.10 |
|  | Kidney | -6.78 ± 0.10 |
| Severe T1DM mice | Heart | -6.70 ± 0.10 |
|  | Liver | -6.55 ± 0.10 |
|  | Spleen | -5.23 ± 0.10 |
|  | Lung | -6.73 ± 0.04 |
|  | Kidney | -6.70 ± 0.15 |
